# Analysis of differences in postural stability in people with adult scoliosis and non specific low back pain

**DOI:** 10.1186/1748-7161-8-S1-P3

**Published:** 2013-06-03

**Authors:** L Bissolotti, V Sani, M Gobbo, C Orizio

**Affiliations:** 1 Servizio di Recupero e Rieducazione Funzionale, Casa di Cura Domus Salutis, Brescia, Italy; 2 Istituto di Fisiologia Umana, Dipartimento di Scienze Mediche e Biotecnologie, Facoltà di Medicina e Chirurgia, Brescia, Italy; 3 LARIN: Laboratorio di Ricerca Neuromuscolare e dell’Attività Fisica Adattata, Italy

## Background

Few papers demonstrated an impairment in postural stability control in patients with non specific low back pain (NL) [1,2]. However, it is not clear whether patients with adult scoliosis (AS) and NL can be considered a specific subgroup.

## Aim

Present a study aimed to compare Postural Stability (PS) in patients with AS and NL[3].

## Methods

Cotrel method was used to assess Cobb angle (CA) on plan x-ray. Using 14 markers, a two optoelectronic infrared cameras (Gemini, BTS spa, Milano, Italy) was used to perform a stabilometric test when patients were keeping a quite standing position with an eyes open trial (EOT), and eyes closed (ECT), and a distance between their feet (FD) as preferred. The Area of Reference Marker on the Ground (C7) (ARMG), Average Marker Speed (AMS) and length of the marker’s trajectory on the ground (LMG) were evaluated during ST.

## Results

AS-Group included 40 patients, 10 men and 30 women, with Cobb angle >15°, mean age 61.8±11.5 years, BMI 23.6±2.8kg/m2. A single curve was present in 32 patients (80%). Cobb angle of primary curve averaged 27.1±11.5° (range, 15–63°), thoracic Cobb angle averaged 25.5±22.3° (range, 8–58°). NL-Group included 40 patients, 9 men and 31 women. Mean age 58.2±10.9 years, BMI 23.9±3.2kg/m2. In AS-group, the self selected mean FD during EOT was 160.1±53.8mm, and during ECT it averaged 160.9±56.2mm (p>0.05). In NL group it was 157.5±53.1mm during EOT, and 154.6±51.2mm during ECT (p>0.05). No differences were noted in both conditions between the two groups (p>0.05). In AS-group, ARMG values averaged 302.6±271.6mm2 during EOT, and 577.9±728.9mm2 during ECT (p>0.05). LMG was 156.9±37.2mm during EOT, and 211.5±72.5mm during ECT (p>0.05); while the AMS was respectively 5.3±1.2mm/sec and 7.1±2.4mm/sec (p>0.05). In NL group, ARMG averaged 296.1±387.6mm2 during EOT, and 876.1±1347.8mm2 during ECT (p>0.05). LMG was respectively 176.1±62.2mm and 246.1±183.5mm (p>0.05); while AMS has been 5.5±1.9mm/sec and 9.9±9.5mm/sec (p>0.05). Romberg Coefficient (RC) was 2.3±1.9 in AS group and 2.9±2.6 in NL group (p>0.05).

## Conclusions

In AS-Group, the ability to control PS with EO and EC was not different than in NL-Group. Physiotherapy program does not require more attention to PS training in AS-Group than NL-Group.
